# 

**Published:** 1875-01

**Authors:** 


					EDITORIAL. ETC.
The Southern Dental Association.?The next Annual Meeting
of this Association will be held in Memphis, Tennesee, on the
9th day of February, 1875, " Marde Gras Day," commencing at
10 o'clock A. M. The Recording Secretary informs us that the
Rail Roads will grant half fare tickets to those visiting Memphis
on the occasion of " Marde Gras."
It is sincerely hoped and expected, that the members of the
following Committees will correspond with each other, so as to
act in concert, and furnish a report upon the subjects on which
they are appointed, and thereby make our next meeting one full
of interest and instruction.
426 Editorial, Etc.
Officers?Dr. J. R. Walker, Louisiana, President; Dr. Isaiah
Forbes, Mo,, 1st Vice President; Dr. W. G. Redman, Ky., 2nd
Vice President; Dr. R. R. Freeman, Tenn., 3rd Vice President;
Dr. Homer Judd, Mo., Corresponding Secretary ; Dr. Jas. F.
Thompson, Va., Recording Secretary ; Dr. J. Hall Moore, Va.,
Treasurer.
Executive Committee?Drs. W. T. Arrington, Memphis, Tenn.;
W. H Morgan, Nashville, Tenn.; Jas. S. Knapp, New Orleans,
La. ; H. J. McKellops, St. Louis, Mo.; S. H. Henkel, Staunton,
Va.
Membership?Drs. S. J. Cobb. Nashville, Tenn. ; C. A. Jordan
Huntsville, Ala.; W. G. Redman, Louisville, Ky.
publications?Drs. R. B. Winder, Baltimore, Md.; J. F.
Thompson, Fredericksburg, Va.; Homer Judd, St. Louis, Mo.
Dental Education?Drs. Homer Judd, St. Louis, Mo. ; H. J.
McKellops. St. Louis, Mo. ; B. F. Coy, Baltimore, Md.
Dental Literature?Drs. W. H. Eames, St. Louis, Mo.: J. P.
H, Brown, Augusta, Ga. ; F. J. S. Gorgas, Baltimore, Md.
Physiology and Surgery?Drs. W. H. H. Thackston, Farmville,
Va. ; J. S. King, Nashville, Tenn. ; A. F. McLain, New Orleans,
La.
Histology and Microscopy? Drs. W. H. Atkinson, New York,
City ; S. P. Cutler, Memphis, Tenn. ; J. H. McQuillen, Phil-
adelphia, Penn.
Dental Chemistry?Drs. J. Taft, Cincinnati, Ohio ; R. Finley
Hunt, Washington, D, 0.; Robt. Arthur, Baltimore, Md.
Pathology?Drs. Isaiah Forbes, St. Louis, Mo. ; Arthur
Ford, Atlanta, Ga. ; E. Floyd, Fayetteville, N. C.
Dental Therapeutics?Drs. W. H. Morgan, Nashville, Tenn.
F. Y. Clark, Savannah, Ga.; R. B. Winder, Baltimore, Md.
Operative Dentistry?Drs. H. J. McKellops, St. Louis, Mo*
J. G. Wayt, Richmond, Va.; J. S. Knapp, New Orleans, La.
Mechanical Dentistry?Drs. Jas. Johnstone, Staunton, Va.
S. Augspath, Helena, Ark.; J. G. McAuley, Selma, Ala.
Voluntary Essays?Drs. W. H. Morrison, St, Louis, Mo. ; J
B. Patrick, Charleston, S. C.; H. B. Noble, Washington, D. C
Dental Appliances and Improvements?Drs. Stellwagon, Phil-
adelphia, Penn. ; Wm. Deasen, Mobile, Ala. ; W. G. Kingsburg,
San Antonio, Texas.
TO READERS AND CORRESPONDENTS.
Communications intended for publication, and exchange journals and papers,
should be addressed to the Editor of the American Journal of Dental
Science, 259 N. Eutaw St., Baltimore, Md. All letters relating to the business
of the journal, or containing cash remittances, to be directed to Messrs. Snowden
& Cowman. Articles for publication should be received by the editor at least
three weeks previous to the first day of the month on which the number in which
it is designed for them to appear, is issued.
f^T~The American Journal of Dental Science will contain tiftj pages of
reading matter in each number, making a volume of not less than
Six Hundred pages
EXCLUSIVE OF ADVERTISEMENTS.
T H Jfi
AMERICAN JOURNAL
OF
DENTAL SCIENCE.
F. J. S. GORGAS, M.D.,D,D.S., Editor.
The Journal is issued on the first of each month and contains not less
than fifty pages of reading matter in each number, exclusive of adver-
tisements, making a volume of six hundred pages per annum.
In each, number will be found Original Communications, Corres-
pondence, Selected Articles, Monthly Summary, Bibliographical No-
tices and Editorial Matter, and every effort will be exerted to render
the Journal worthy of the. patronage of the Dental profession.
A generous co-operation is Respectfully solicited from the members
of the profession ; and as the Journal has now a printing office of its
own which materially lessens the cost of its publication, it will be
issued at the reduced subscription price of
!.SO For Annum in A.tiLvanoe.
Communications are respectfully solicited on all subjects pertain-
ing to the practice of dentistry, as well as reports of cases occurring
in dental practice;
RATES OF ADVERTISING.
1 Page.
I "
k " ?
1 MO.
$15 00
10 00
7 00
5 00
2 MO.
$22 50
15 00
n oo
'8 00
3 MO.
$30 00
20 00
15 00
11 00
6 MO.
$50 00
30 00
20 00
15 00
12 MO.
$100 00
50 00
30 00
20 00
All original communications designed for publication, works for
review, new instruments for notice, exchanges, &c., should be directed
to the editor, 259 N. Eutaw St., Baltimore, Md.
All advertisements and subscriptions should be addressed to
SNOWDEN & COWMAN,
Publishers of the Am. Journal of Dental Science.
86 W. Fayette St. Bet. Charles & Liberty Sts.,
BALTIMORE, Md

				

